# E-Health implementation in Pakistan: challenges, opportunities, and the path forward

**DOI:** 10.3389/fdgth.2026.1747740

**Published:** 2026-04-29

**Authors:** Amjad Islam Amjad, Sarfraz Aslam

**Affiliations:** 1 Department of Education, The University of Lahore, Lahore, Pakistan; 2 Faculty of Education and Humanities, UNITAR International University, Petaling Jaya, Malaysia

**Keywords:** artificial intelligence, digital, e-health, healthcare, medical issues

## 1 Introduction

Despite global progress, digital health adoption in low-income countries remains uneven. Pakistan presents a critical case due to its large population, rural-urban disparities, limited public health expenditure, and emerging yet fragmented digital initiatives. While global trends show a shift towards E-Health, Pakistan presents a unique case study where these global innovations meet specific local implementation barriers (1).

The World Health Organization (WHO) has defined E-Health as the service provided for healthcare using ICT platforms (2). E-Health includes ICT applications that directly impact patients' care (3). E-Health primarily refers to the application of computer- or internet-based technology, whereas telehealth primarily involves telephone- or video-based technology (4). In this paper, the term E-Health is used as an umbrella encompassing telehealth, digital medical records, data-driven systems, and AI-enabled services. E-Health has the potential to transform the healthcare system (5).

The developed countries allocate funds to promote innovative E-Health systems to prioritize the most efficient methods of providing top-notch healthcare services to their citizens (6). For example, Germany has implemented E-Health cards for insured people that contain confidential information, including insurance details and medical records. Patients use “smart cards” to access healthcare treatments covered by their insurance (7). Similarly, the USA, UK, Australia, and Canada have also introduced E-Health initiatives to promote immediate and quality patient healthcare (8, 9).

However, underdeveloped countries face problems adopting E-Health programs (10). Pakistan is an important country in the South Asian region, where E-Health is partially implemented (11). Pakistan is now committed to partnering with international organizations to develop a tech-driven system to improve its healthcare and delivery (12). Pakistan has a doctor-to-patient ratio of approximately one doctor per one thousand people (13). This also calls for the urgent need for E-Health initiatives to meet healthcare needs.

In this manuscript, we use the Technology Acceptance Model (TAM) (14), the Diffusion of Innovations (DOI) (15), and Human-Centered Design (HCD) (16) as theoretical frameworks to understand E-Health technology adoption and diffusion in Pakistan. TAM helps us understand how users' attitudes towards technology influence acceptance or rejection of technology. DOI helps us understand how technology diffusion occurs in society, especially in rural areas. HCD also helps us understand that users are at the center of any design and how this plays out in E-Health technology, where user-centered design is a significant factor in its success in Pakistan. These models provide us with a framework for understanding how E-Health technology adoption and diffusion are possible in Pakistan, especially in its unique sociotechnical context.

This paper adopts a critical and interpretive perspective to examine the E-Health landscape in Pakistan, arguing that digital health transformation is not merely a technological shift, but a sociotechnical process shaped by structural inequalities and institutional constraints. Drawing explicitly on the TAM, DOI, and HCD, the paper analyses how adoption barriers, diffusion limitations, and user-centered design gaps interact to shape implementation outcomes, advancing a stakeholder-enabler perspective. This paper will contend that although E-Health is vital for enhancing healthcare services in Pakistan, it faces challenges, including infrastructure, governance, and literacy issues. These challenges hinder the adoption of E-Health technology, despite its potential to fill the gaps in healthcare services.

## 2 Digital transformation and emerging care models

However, the digital transformation in the healthcare sector is not only about technological shifts but also about the restructuring of healthcare service delivery models, especially in Pakistan's resource-constrained environment. From the DOI perspective, the digital platform facilitates the diffusion of healthcare in new forms beyond institutional boundaries; however, TAM emphasizes the importance of perceived usefulness and ease of use in the adoption of healthcare services (17). However, these benefits remain contingent on contextual adoption conditions. In Pakistan, structural inequalities and digital divides limit the realization of these efficiencies, suggesting that technological potential does not automatically translate into systemic impact.

It can also help medical institutions better understand patients' needs and conditions, allocate medical resources more accurately, and improve the pertinence and effectiveness of medical services (18). It can also enhance management levels and efficiency through data analysis and intelligent management, as well as predict disease occurrence and development trends through data monitoring and analysis. Mobile phones account for more than half of all Internet traffic, primarily used for communication, research, and financial transactions. Because billions of people worldwide have access to the Internet, it is now easy to provide healthcare at the patient's convenience (19). Compared with progress in other industries, the healthcare booking process remains a significant challenge. Patients dial into the clinic, and operators manually book them into slots.

In Pakistan, emerging on-demand healthcare models, including Sehat Kahani, DoctHERs, and Teeku Telehealth, illustrate the early-stage diffusion of digital health innovations, albeit unevenly across regions and populations. This uneven diffusion reflects classic DOI patterns, where early adoption remains concentrated among more accessible and digitally literate populations, potentially reinforcing existing healthcare disparities. It is pertinent to note that most female physicians at Sehat Kahani with 6-10 years of experience are satisfied with on-demand healthcare provision (20). Similarly, a pilot project on DoctHERs also yielded satisfactory results, meeting the demand for on-demand healthcare (21). This suggests that most people in developing countries, such as Pakistan, are ready to adopt telehealth initiatives, like Teeku (22). Thus, telehealth providers can be approached using easy tools to meet patient healthcare needs.

## 3 Implementation of E-health in Pakistan

### 3.1 Governance, policy, and operational landscape

From a governance perspective, Pakistan’s E-Health landscape reflects a misalignment between policy ambition and operational execution. Pakistan has articulated strong policy intentions toward E-Health development, particularly through the National Digital Health Framework 2022–2030. The government is interested in the digital transformation of the healthcare system in remote areas of Pakistan. This shows that fragmented healthcare delivery, low public health expenditure, and workforce shortages require systemic technological support (23). Beyond national policy commitments, private organizations and NGOs are working on several E-Health projects. These projects indicate partial operationalization of E-Health services. However, these projects vary in scale, governance structure, and technological usage in remote areas (24). Qureshi (25) mentions that a considerable proportion of Pakistan's population lacks access to basic digital health infrastructure, reinforcing structural inequities in healthcare delivery. This calls for the urgent need for E-Health.

**Figure 1:**
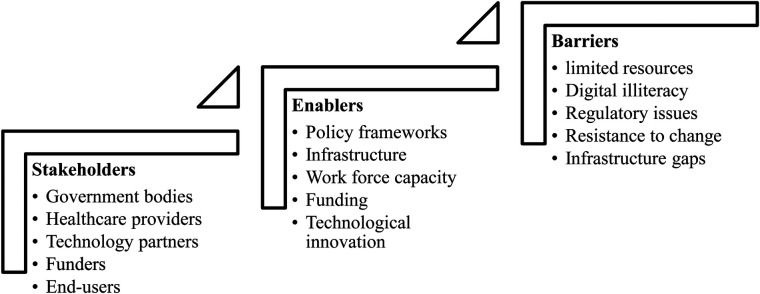
This figure represents the various stakeholders, systems, and factors involved in the implementation and adoption of E-health in Pakistan.

Figure 1 illustrates the key stakeholders involved in the implementation of E-Health in Pakistan, including government bodies, healthcare providers, technology developers, patients, and private sector partners. Using the TAM, DOI, and HCD frameworks, we see that these stakeholders influence the adoption and diffusion of E-Health. Government support is crucial in creating favorable conditions for technology adoption, while healthcare providers and patients must perceive the technology as useful and easy to use, as highlighted by TAM. The DOI framework explains how innovation spreads unevenly, with early adopters driving the process, while HCD ensures that the solutions meet user needs, facilitating broader acceptance and integration.

### 3.2 Challenges to the implementation in Pakistan

Several interconnected structural and systemic barriers limit E-Health implementation in Pakistan, which can be critically interpreted through TAM, DOI, and HCD frameworks. For example, the digital infrastructure gap is a major limitation. Rural and remote areas had uneven internet connectivity. Limited internet connectivity and power supply issues can limit consistent use of digital platforms, particularly in remote areas. In addition, several public health facilities lack interoperable health information systems, which hinders integration and scalability. From a DOI perspective, uneven infrastructure constrains the diffusion of innovation, particularly in rural areas where technological access remains limited.

Besides infrastructure, workforce, and capacity constraints, other constraints also hinder E-Health implementation in Pakistan. There is a shortage of healthcare professionals trained in digital health technologies. Practitioners lack formal exposure to electronic health records, teleconsultation systems, and data-driven decision tools (26). The implementation of E-Health cannot be achieved without structured training and institutional incentives. This reflects TAM-related barriers, where low perceived ease of use and limited digital competence reduce technology acceptance among healthcare providers.

Moreover, governance and regulatory challenges may also slow the process. Although national policies have been proposed, operational guidelines, enforcement mechanisms, and standardized protocols remain underdeveloped. However, operational guidelines, enforcement mechanisms, and standardized protocols are still evolving. The lack of regulatory steps may also reduce coordination among agencies. From an HCD standpoint, weak regulatory alignment indicates a lack of user-centered policy design, limiting system responsiveness to stakeholder needs.

### 3.3 Strategic opportunities and future directions

Despite structural constraints, E-Health in Pakistan presents significant strategic opportunities, particularly when examined through the lens of HCD and DOI, which emphasize user-centered scalability and adaptive innovation pathways. For example, Sehat Kahani connects patients with female doctors via mobile applications and e-clinics, whereas DoctHERs offers affordable healthcare via Skype and mobile platforms. However, they require integration into the national policy framework and funding support. This indicates that without coordinated institutional integration and sustained funding mechanisms, such initiatives risk remaining isolated rather than systemically transformative.

Data collection and the integration of AI in healthcare have changed prescribing practices, enhanced preventive programs, and facilitated resource allocation. The potential of AI in medical research, such as precision medicine and drug development, is beneficial but is accompanied by concerns about ownership, transparency, and ethics in AI applications in healthcare. Thus, the implementation of AI in E-Health projects needs to be carefully considered to avoid widening healthcare inequalities. Without careful governance, AI integration may reproduce existing inequities, privileging digitally literate populations while marginalizing vulnerable groups. Digital transformation has emerged as the most effective solution to address the imbalance between medical resources and needs in Pakistan. The E-Health sector has a bright future in improving healthcare and increasing the accuracy of results (27). Presently, there exists a deficiency in the understanding and execution of E-Health in Pakistan (28).

Future progress requires three coordinated priorities to improve infrastructure and healthcare delivery. Future progress deregulation and interdependent priorities: strengthening digital infrastructure with a focus on rural inclusion, developing robust governance frameworks for data protection and AI regulation, and enhancing digital literacy among both healthcare providers and patients. These priorities must be implemented in an integrated manner to avoid reinforcing existing systemic inequalities.

## 4 Conclusions

E-Health in Pakistan should not be understood as a purely technological advancement but as a complex sociotechnical transformation that requires critical alignment among policy, practice, and user realities. Critically, without addressing underlying governance fragmentation and sociotechnical divides, E-Health initiatives risk becoming technologically advanced yet socially exclusionary. Sustainable technological transformation requires coordinated policy leadership, ethical oversight, stakeholder alignment, and targeted investment in digital equity. This paper emphasizes the need to strategically integrate E-Health policies and technologies in Pakistan. Overcoming these barriers requires coordinated efforts from government, healthcare providers, and the private sector to enhance healthcare access and efficiency.
